# Semantic Dementia without Surface Dyslexia in Spanish: Unimpaired Reading with Impaired Semantics

**DOI:** 10.3233/BEN-2012-119009

**Published:** 2012-03-19

**Authors:** Maximiliano A. Wilson, Macarena Martínez-Cuitiño

**Affiliations:** ^1^Centre de recherche de l’Institut universitaire en santé mentale de Québec (CRIUSMQ)Département de réadaptationUniversité LavalQCCanada; ^2^Institute for Cognitive Neurology (INECO) and University of Buenos AiresBuenos AiresArgentina

**Keywords:** Semantic dementia, surface dyslexia, reading

## Abstract

Surface dyslexia has been attributed to an overreliance on the sub-lexical route for reading. Typically, surface dyslexic patients commit regularisation errors when reading irregular words. Also, semantic dementia has often been associated with surface dyslexia, leading to some explanations of the reading impairment that stress the role of semantics in irregular word reading. Nevertheless, some patients have been reported with unimpaired ability to read irregular words, even though they show severe comprehension impairment. We present the case of M.B., the first Spanish-speaking semantic dementia patient to be reported who shows unimpaired reading of non-words, regular words, and–most strikingly–irregular loan words. M.B. has severely impaired comprehension of the same words he reads correctly (whether regular or irregular). We argue that M.B.’s pattern of performance shows that irregular words can be correctly read even with impaired semantic knowledge corresponding to those words.

## 1. Introduction

Surface dyslexia has been described as arising from an overreliance on the phonological or sub-lexical route for reading [15,23,35,49,50]. Typically, acquired surface dyslexic patients show spared reading of non-words and regular words. They also show a regularization effect. That is to say, their reading of regular words is generally better than their reading of irregular (or exception) words. Thus, they have the tendency to "regularize" irregular words (IWs) because they read them through grapheme-phoneme conversion (GPC) rules.

Figure 1 shows a dual-route reading model [8,9]. Within this model, damage to more than one component may cause patients to read via GPC rules (route 1, Fig. 1). For example, damage to the orthographic input lexicon would prevent reading via routes 2 and 3, forcing the patient to rely on the sub-lexical route for reading. Then, she would show unimpaired non-word and regular word reading. Conversely, her ability to read irregular words would be impaired and regularisation errors should occur. This sub-type of surface dyslexia has been called "input" surface dyslexia [14,15].

Also, damage to the phonological output lexicon would impair patients' ability to retrieve the spoken form of words, causing the "output" sub-type of surface dyslexia [14,15]. Though forced to use the GPC rules to read words, they can recognize words and access their meaning (i.e., word-picture matching and lexical decision tasks would be unimpaired), as routes 2 and 3 work partially (see Fig. 1). This kind of patient would fail in oral picture naming tasks and would commit regularisation errors when reading words aloud.

Most of the surface dyslexic patients have been reported in opaque orthographies (that is to say, languages with inconsistent orthography-phonology map-

ISSN 0953-4180/12/$27.50 © 2012 – IOS Press and the authors. All rights reserved

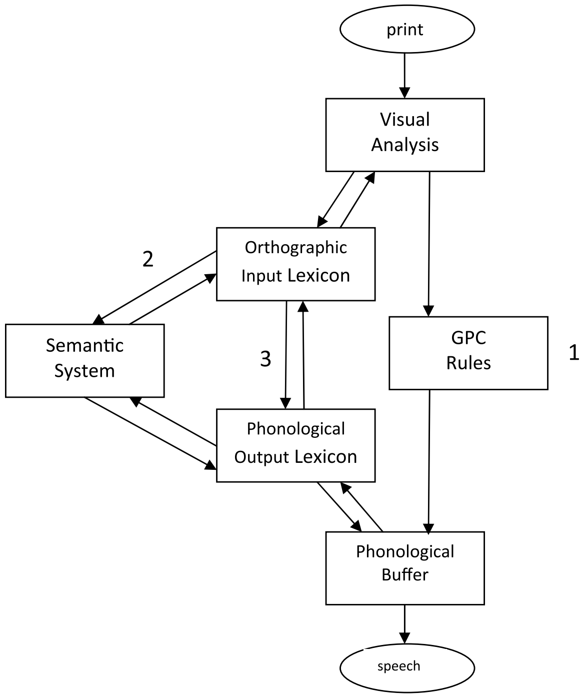

Fig. 1. Routes for reading aloud in a dual-route model: 1) Non-lexical; 2) Lexical semantic route; 3) Lexical non-semantic route (adapted from Coltheart et al. [7], with permission of the authors).

pings) such as English [1,14,20,50] or in an ideographic orthography such as Japanese [21,45]. However, the existence of two routes (lexical and sub-lexical) for reading in transparent orthographies (languages that have highly consistent orthography-phonology mappings) has been strongly indicated by the report of both phonological and surface (input and output types) dyslexic patients in this kind of languages (in Spanish [12,17–19,31,36]; in Italian [4,22,32,37,44,51]).

In addition to the input and output types of surface dyslexia, many patients with semantic impairment due to diffuse lesions caused by dementia (of both the Semantic and Alzheimer's types) also suffer from Surface Dyslexia. This has led some authors to stress the relationship between semantic impairment and reading impairment [24,41,53]. These authors highlight the importance of unimpaired semantics for reading, especially for irregular low-frequency words see [20]; also [24, 41].

However, Schwartz et al. [46,47] reported the case of a patient, W.L.P., who showed unimpaired non-words, regular and irregular word reading yet suffered from anomic dementia indicative of a semantic impairment. Moreover, as W.L.P.'s understanding of words deteriorated, her continuing presentation of intact reading suggested that her reading abilities could not be semantically mediated [47, p. 264]: She was severely anomic and failed in semantic categorisation tasks. Schwartz et al. claimed that since W.L.P. did not regularize the IWs she read correctly (thus, she did not seem to apply GPC rules), this would imply that she used a direct whole-word print-to-sound association route instead of GPC rules.

Our focus will be on the dependence or independence of semantic knowledge and reading highlighted by cases such as that of W.L.P. A number of other patients have been reported, following W.L.P., who could also read IWs with comprehension impairments [7,23, 33,34,43]. Their pattern of performance has been taken as evidence in favour of the existence of a third route for reading that implies the direct conversion of entire words from print to sound, bypassing semantics. In other words, a direct route from the orthographic input lexicon to the phonological output lexicon without the intervention of the semantic system (route 3, Fig. 1).

Some patients with dementia but without surface dyslexia have also been described in transparent languages. In 1989, Carlomagno et al. [5] described an Italian-speaking patient, I.R., who suffered from reduced language comprehension but fluent and anomic language production. A CT scan showed left temporal atrophy. He failed in semantic tasks but could read non-words and words almost perfectly. When reading words he committed only 17% of stress regularisation errors – it is noteworthy that the only feature in Italian that cannot be derived from orthography is syllabic stress and it has to be assigned through lexical look-up [3,4]. Carlomagno et al. concluded that patient I.R. could only read words with irregular stress correctly, given his semantic impairment, through a direct non-semantic route, in an account homologous to the explanation of W.L.P.'s preserved irregular word reading [46,47]. A similar patient who suffered from semantic dementia yet could read non-words, words and irregular loan words with impaired semantic knowledge has been described in Portuguese. Hosogi Senaha et al. [28] studied patient L.G.S. who had fluent but anomic language production and comprehension difficulties. Her MRI scan showed bilateral temporal atrophy more marked on the right lobe. They also concluded that this patient had a direct lexical non-semantic reading that enabled her to read IWs correctly. To the best of our knowledge no patient with semantic dementia and unimpaired IW reading has been reported in another transparent language, such as Spanish.

Spanish is a very consistent language for reading but this does not necessarily mean that it is perfectly transparent. As a general rule, one grapheme corresponds to one phoneme. In spite of this, some exceptions to these one-to-one correspondences can be found. For instance, there are five digraphs in Spanish – two graphemes that correspond to one phoneme: "ch", "ll", "qu", "gu", and "rr". Also, the pronunciation of two graphemes is contextually dependent because it depends on the vowel that follows them. These two graphemes are "c" and "g" and they are pronounced /θ/ (as in "celeste") or /x/ (as in "general"), respectively, if followed by "e" or "i", but their pronunciation is /k/ (as in "casa") or /g/ (as in "gato"), respectively, if followed by any of the three remaining vowels [10]. In addition, the pronunciation of the grapheme "r" depends on its position in the word, for example, if it is at the beginning of a word it is pronounced as the digraph "rr" /r/ (as in "rosa") and if it is in any other part of the word it is pronounced /?/ (as in "cara"). Finally, the grapheme "h" is a silent letter, it is not pronounced at all.

Not all varieties of Spanish have the same number of phonemes. In the version of Spanish spoken in Argentina, also called the Rio de la Plata variety, there are 22 phonemes [18]. In this version, the graphemes "z" and "s" are both pronounced /s/ (for example, "seta" and "zeta" are homophones) and the grapheme "c" is also pronounced /s/ if followed by "e" or "i" (thus, the words "ciento" and "siento" are homophones).

Most critically for our concerns, in addition, the Spanish spoken in Argentina has kept the original orthography and phonology of foreign loan words. Argentina has a long tradition of polyglotism and this influence is evidenced even now in currently standard pronunciation of foreign words [6]. These words have kept their original orthography and phonology. Thus, foreign words like "shampoo" are pronounced exactly as in English (\sham-'pü\). In contrast, the standard accepted pronunciation in the Spanish of Spain would render the word "shampoo" in the regularized form "champú" that makes the phonology of the foreign word more consistent with Spanish orthography. Importantly, these loan words are very frequently encountered and used in Argentina [18].

We were able to exploit the presence of foreign loan words in Rio de la Plata Spanish to examine the dependence or independence of semantic knowledge and the reading of regularly or irregularly pronounced words. While Spanish is predominantly a language with a transparent orthography, the adherence to original pronunciations of foreign loan words in Argentina means that such words truly constitute irregular words. The aim of our article is to present the first case of a Spanish-speaking semantic dementia patient who had unimpaired non-word, word and IW reading with comprehension impairment.

## 2. Case report

### *2.1. Background and general neuropsychological assessment*

M.B. is a 69-year old right handed male with 12 years of education who was diagnosed with semantic dementia. His first symptoms were comprehension difficulties and anomia. He had problems in recalling people's names and common words that he could not understand. He also began to demonstrate problems in understanding the content of newspapers which he used to read every day. He could read words but without understanding them.

In addition, he evidenced mildly impaired writing. When tested with a spelling to dictation task [52] he showed a tendency to write words with homophone graphemes for example, "casa" (house) instead of "caza" (hunting), which – as mentioned before- are homophones in Argentine Spanish. He did not commit errors of omission or addition of graphemes.

Conversely, M.B.'s episodic memory was preserved and he could remember details of his everyday activities. His working memory was quite intact: 5 and 4 digits, forward and backwards, respectively. His spontaneous speech was fluent with normal articulation and syntax but severely anomic.

In sum, at the moment of assessment he presented a 7-year progressive loss of vocabulary and comprehension impairment. The CAT scan (see Fig. 2) revealed bilateral temporal lobe atrophy, more lateralised to the left temporal lobe. It also showed atrophy of the anterior portions of the temporal lobes [42].

## 3. Experimental investigations

In this section the performance of M.B. in different tasks that assessed lexical, sublexical and semantic processing will be reported. The first set of experimental tasks assessed M.B.'s sublexical and lexical reading routes by means of non-word, word, and IW reading tasks. The second group of tests explored word recognition, specifically the input orthographic lexicon through visual lexical decision tasks and an IWs phonology-to-orthography matching task. The third set

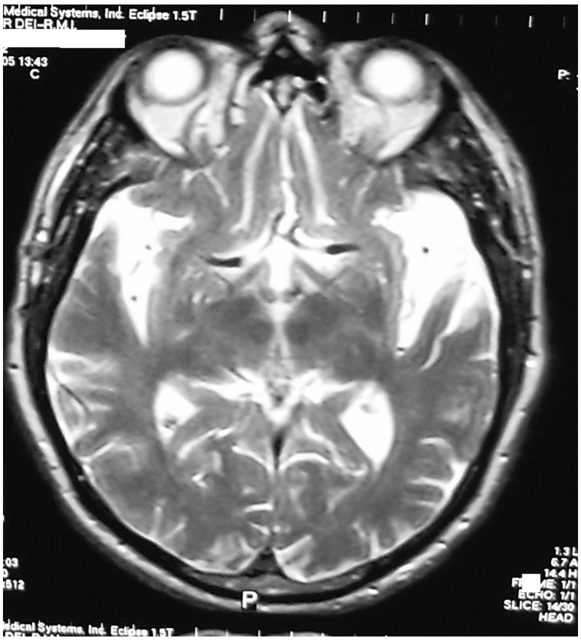

Fig. 2. Patient M.B.'s CAT scan that shows bilateral atrophy of the temporal lobes.

of tasks assessed M.B.'s semantic processing through a variety of different tests, such as picture naming or word-picture matching. If M.B.'s performance on regular and irregular word reading shows no difference with that of normal control subjects but his performance on semantic tasks is impaired then semantic mediation could not be considered of capital importance for reading – especially IWs [40,41,53].

### 3.1. *Reading*

Unimpaired non-word reading has usually been associated with the correct application of GPC rules. Also, unimpaired (regular) word reading in a transparent language like Spanish can be achieved via GPC rules. However, IW reading could only be achieved through lexical reading, otherwise regularisation errors should occur. In this section the tasks that assessed M.B.'s reading abilities will be presented.

#### 3.1.1. *Non-word reading*

This task consisted of 20 legal pronounceable nonwords taken from the *Batería de Evaluación de la Afasia* (BEA, Battery for the Assessment of Aphasia) [52]. Non-words varied in their length (from 1 to 4 syllables) and in their syllabic complexity. M.B. read correctly 19/20 (95%) non-words, with no significant differences compared to the mean performance of normal controls (mean score = 95%; $\chi^2$ (1) = 0.36, $p$ = 0.54).

This result implies that M.B. could apply GPC rules normally. Although some authors [34,39] claim that a patient with unimpaired sublexical processing would not constitute a "pure" case of a non-semantic reader, M.B.'s unimpaired use of GPC rules is in line with patients E.W. [23] and W.L.P. [46,47] who, also, could read non-words. In addition, Ellis et al. [14] consider damage to the lexical semantic route as the locus of impairment for lexical non-semantic reading, without additional impairment of the sub-lexical route.

#### 3.1.2. *Regular word reading*

M.B. was administered the Spanish adaptation of the 64-Semantic Battery (64-SB) [2,25] that consisted of reading, spelling to dictation, picture naming, and word-picture matching tasks with the same 64 nouns, divided into different semantic categories (i.e., animals, household items, tools, etc.).

In word reading, M.B. read 62/64 (97%) words correctly, showing no significant difference with a normal control group (mean score = 64; Fisher's exact $\chi^2$ (1) = 2.03, $p$ = 0.49). Both his errors were in the category "animals": He mispronounced "Águila" (eagle), which in Spanish should be stressed in the (accent marked) antepenultimate syllable, saying instead "aGUIla" (incorrectly placing the stress on the penultimate syllable, the stressed syllable is in uppercase). And he committed a visual error by reading "rana" (frog) as "rama" (branch).

#### 3.1.3. *Irregular word reading*

So far, M.B. could simply be characterized as a nonlexical reader. In other words, since Spanish has very consistent orthography-phonology mappings his unimpaired reading of words and non-words could be explained by application of GPC rules. He is able to read non-words and words that in Spanish are almost completely regular and thus can be derived directly by applying GPC rules. As stated before, the version of Spanish spoken in Argentina maintains the irregular orthography-phonology correspondences of foreign loan words so that such words, which are in common usage, can be said to have irregular pronunciations. Hence, if M.B. can read these irregular words (IWs) correctly, we would find evidence for lexical processing in reading aloud.

We selected the irregular words for use in the reading test from a candidate set rated on familiarity. Since the existing frequency databases for Spanish -for ex-

Table 1
Patient M.B. and controls' performance on the reading tasks

| | MB | | Controls | | |
|---|---|---|---|---|---|
| Tasks | Score | % | Score | % | Sign. |
| Non-words (BEA) | 19/20 | 95 | 19/20 | 95 | ns |
| Regular words (64-SB) | 62/64 | 97 | 61/64 | 95 | ns |
| Irregular words | 39/40 | 98 | 39/40 | 98 | ns |

ns = non significant.

ample, the LEXESP database [48] – do not have entries for IWs, we collected familiarity ratings for a set of 225 IWs currently used in Argentina. Twenty-four subjects, native speakers of the Argentine variation of Spanish were asked to judge the degree to which they came in contact with or thought about each one of the selected words. A 7-point scale that ranged from "very unfamiliar ("1") to very familiar ("7") was used [10]. A set of 40 irregular words was chosen from the list of 225 (see Appendix A). Half the items were highly familiar (mean = 5.12) and half the words were of low familiarity (mean = 2.83). Both familiarity groups were matched for grammatical category (all nouns), bigram frequency (token and type), length in letters, and neighbourhood size (N-size).

M.B. read 39/40 (98%) irregular words correctly and his performance did not differ from that of normal controls (mean score = 39; sd = 0.51). M.B. only misread the word "panaché", regularising the "ch" sound. Additionally, neither normal controls nor M.B. showed an effect of familiarity in their performance ($p > 0.1$).

Table 1 shows M.B. and controls' score and percentage of correct responses in the three reading tasks. It can be seen that M.B. achieved almost perfect scores in all the tasks, regardless of the type of item: non-words, regular words or irregular words. This suggests that both his sub-lexical (non-words) and lexical (regular and irregular words) routes for reading were unimpaired.

### 3.2. *Visual word recognition*

In this section, our assessment of M.B.'s orthographic input lexicon will be presented, which was conducted particularly by means of a lexical decision task in which the stimuli included words and pseudohomophones. This task constitutes a very demanding test of the preservation of M.B.'s orthographic lexicon. Additionally, the relation between the phonology and orthography of IWs will be explored with a matching task.

#### 3.2.1. *Lexical decision*

M.B. was administered a lexical decision task taken from the BADA Battery [16,38]. This task consisted of 40 words and 40 pronounceable legal non-words. M.B. achieved a score of 79/80 (99%) correct responses, placing his performance on this task within normal limits [16]. His only mistake was a false positive: he accepted a non-word as a word.

#### 3.2.2. *Lexical decision with pseudohomophones*

In order to further assess M.B.'s orthographic input lexicon, we administered a more complex lexical decision task that included pseudohomophones [18,36]. Pseudohomophones are non-words with a phonology that corresponds to an existing word but an orthography that does not exist in the lexicon, for example, "paisage" (landscape), is correctly written as "paisaje" in Spanish but both "g" (followed by "e" or "i") and "j" are pronounced /X/. The stimuli in the task consisted of 60 nouns (half high- and half low-frequency), 30 legal pronounceable non-words, and 30 pseudohomophones. Given the phonological identity of pseudohomophones with real words and the orthographic similarity of these non-words to the real words with which they were homophonous, this task imposes greater demands on the orthographic input lexicon. If M.B.'s performance in this task is also unimpaired, it will provide stronger evidence of the correct functioning of his orthographic lexicon. The patient's responses and reaction times (RTs) were recorded. RTs were measured from stimulus onset to response onset.

All three stimulus groups (words, non-words, and pseudohomophones) were matched for word frequency (the frequency of the original words from which the non-words and pseudohomophones were derived was used here), bigram frequency (token and types), length (in number of letters, syllables and phonemes), and neighbourhood size (N-size). N-size and frequency (lexical and bigram) values were taken from the B-Pal database [13].

M.B. obtained a score of 118/120 (98%) correct responses, a score that is actually above the mean score of normal controls matched for schooling and age (mean score = 112, sd = 4.65). Nevertheless, this difference was not statistically significant (Yates' corrected $\chi^2$ (1) = 2.61, $p = 0.10$). M.B.'s two mistakes were made in accepting a non-word as a word, and a homophone non-word as a word.

Regarding the patient's RTs, a one-way ANOVA with type of stimulus (word, non-word, and pseudohomophone) as factor showed that the difference between words (mean = 870 ms), non-words (mean = 1168 ms), and pseudohomophones (mean = 2377) was highly significant (F [2, 117] = 91.07, $p < 0.001$). Tukey's HSD post-hoc comparisons showed that all three groups were significantly different from each other ($p < 0.05$). When compared to those of normal controls (mean = 1250 ms), M.B.'s RTs (mean = 1322 ms) were not significantly longer (t (238) = 0.90, $p = 0.37$).

This pattern of performance is in line with that found by Cuetos and Domínguez [11] in normal subjects. In order to decide whether a pseudohomophone is a word or not larger RTs are expected to pseudohomophones than to other kinds of non-words because it is easier to reject a non-psuedohomophonous non-word in comparison to a pseudo-homophone since the latter is very similar, orthographically and phonologically, to a real word.

If we consider only the words, M.B. showed an effect of frequency: his RTs for high frequency (mean = 712 ms) were significantly lower than his RTs for low frequency words (mean = 1028 ms; $t$ (58) = −2.88, $p < 0.01$).

### 3.2.3. Irregular word phonology – Orthography matching task

This matching task assessed M.B.'s phonology-to-orthography mappings for IWs. Following a similar procedure to that used by Blazely et al. [1], the patient heard the spoken pronunciation of the IWs and was asked to match them with their correct orthography. The 40 IWs from the IW oral reading task were used. The patient was asked to match the pronunciation of each IW to their spelling, choosing between the correct orthography and a regularized orthographically legal alternative. For example, for the item "bouquet" we also provided the pseudohomophone spelling "buqué", which gives what would be the regular orthography for the sound of the target word. Both groups of orthographic alternatives were matched for bigram frequency. If M.B. processed the phonology of the IWs via sublexical processes, then he would prefer the regularized pseudohomophone orthography. Conversely, if he directly linked the whole word phonology to its orthography he would accept its correct irregular orthography. A pattern of the first type would provide evidence for a competing sublexical alternative which is also coherent with the orthography of the majority of the words in Spanish.

M.B. correctly matched the phonology with the orthography of 37/40 (93% correct) IWs, showing no significant differences with the performance of normal controls (mean score = 38, sd = 1.42; $\chi^2$ (1) = 0.21, $p > 0.1$). Additionally, he did not show an effect of familiarity. He matched correctly 18/20 (90%) high familiarity words and 19/20 (95%) low familiarity words ($\chi^2$ (1) = 0.36, $p > 0.1$).

Table 2 shows a summary of M.B. and controls' performance and RTs in the lexical decision and the IW orthography-to-phonology matching tasks. In summary, M.B. showed an effect of lexicality (shorter RTs for words than for non-words whether homophones or not) and an effect of frequency (shorter RTs for high frequency words as compared to low frequency ones), and near perfect scores in both tasks. These results strongly suggest that M.B.'s orthographic input lexicon was unimpaired and functioned within normal limits. Additionally, his performance in the matching task was also unimpaired suggesting that he used direct connections between the phonology and the orthography of IWs.

## 3.3. Semantics

The following set of tasks assessed the contribution of semantics to M.B.'s reading performance, claimed by some authors to be crucial for reading irregular words [40,41,53].

### 3.3.1. Picture naming

M.B.'s naming of the 64 nouns of the 64-Semantic Battery [2,25] was severely impaired. He named correctly only 18/64 (28%) pictures. M.B.'s score on picture naming was significantly lower in comparison with that of normal controls (mean score = 64; Fisher's exact $\chi^2$ (1) = 71.80, $p < 0.001$). His errors were mostly anomias (41/46, 89% of the total errors) and on five occasions (11%) M.B. produced a semantically related word: horse, instead of camel; dog for tortoise; rat for mouse; banana instead of orange; car for lorry; and plane instead of helicopter.

### 3.3.2. Word-picture matching tasks

M.B. was also administered a written and a spoken word-picture matching tasks taken from the BEA Battery [52]. The patient was asked to match the written -or spoken- names of 20 object nouns to their corresponding pictures. For each target picture there were three distractors: A semantically related picture, a phonologically related one and a non-related one.

In the written version M.B. correctly matched 11/20 (55%) names to their corresponding pictures. In the spoken matching task, M.B. matched 12/20 (60%)

Table 2
Patient M.B. and controls' performance and RTs on the lexical decision (LD) and irregular word (IW) phonology-orthography matching tasks

| | MB | | | | Controls | | | | |
|---|---|---|---|---|---|---|---|---|---|
| | Accuracy | | RTs (in ms) | | Accuracy | | RTs (in ms) | | |
| Tasks | Score | % | Mean | SD | Score | % | Mean | SD | Sign. |
| Lexical decision (BADA) | 79/80 | 99 | – | – | 79/80 | 99 | – | – | ns |
| LD with pseudohomophones | 118/120 | 98 | 1322 | 799 | 112/120 | 93 | 1250 | 325 | ns |
| IW Phonology–Orthography matching | 37/40 | 93 | – | – | 38/40 | 94 | – | – | ns |

ns = statistically non significant.

words to their correct pictures. All of his errors were semantically related to the target, except for two that were phonologically related ones. His performance was significantly below that of the control group in both tasks (mean score for both tasks = 20; Fisher's exact $\chi^2$ (1) = 11.61, $p < 0.01$, for the written version; Fisher's exact $\chi^2$ (1) = 10.00, $p < 0.01$ for the spoken version).

### 3.3.3. *Pyramids and palm trees test*

The previous results can be interpreted as indicating impaired access to semantics from pictorial and lexical inputs. However, an alternative interpretation would be that the observed performance stemmed from a central semantic impairment. In order to obtain further evidence in favour or to rule out the second explanation, M.B. was also administered the Pyramids and Palm Trees test [30]. This is the most widely used test for semantic association and it consists of 52 pictures or words that the patient has to match to their closest semantically related picture or word out of two options.

M.B. associated 22/52 (42%) correctly for the pictorial modality and 20/52 (38%) for the verbal modality. As Howard and Patterson [30] suggest, when impairment is found in both modalities of access to semantics, then a lexical-semantic impairment can be inferred. That is to say, it is not the access modality -from pictures to semantics or from words to semantics- that is impaired but the semantic system itself. These results are in line with the previous tests and seem to confirm the hypothesis of a central semantic impairment.

### 3.3.4. *Picture semantic categorisation*

We further tested semantic knowledge using a picture semantic categorisation task taken from the 64-Semantic Battery [2,25]. The patient was asked to match the picture of an object to its category by choosing from eight different written categories (for example, birds, animals, means of transport, tools, etc.).

M.B. obtained 50/64 (78%) correct responses. His score was significantly lower than that of normal controls (mean score = 63; sd = 0.196; $\chi^2$ (1) = 12.76, $p < 0.001$). The patient demonstrated difficulties in correctly identifying the elements of six (out of eight) categories. He showed unimpaired knowledge of the categories "fruit" and "means of transport".

### 3.3.5. *Homophone comprehension task*

In order to further assess his access to semantic knowledge, M.B. was administered a homophone comprehension task [18,36]. In this kind of task, it is the correct mapping between orthography and its precise semantic correspondence that helps subjects disentangle if the presented definition corresponds to the target word or to a possible competitor (i.e., its homophone). If M.B. performs poorly in this task, then it would be in line with the results found in the other semantic tasks and would provide further evidence for the central semantic impairment explanation for M.B.'s difficulties.

In this version of the task with 28 items, patients are presented with a word printed on a card and a written definition. They are asked to say whether this definition corresponds to the printed word. For half the trials ($n = 14$), the visually presented definition corresponds to the target printed word, and in the other half, to its homophone match. In a second session, the same target words are presented but where previously the word had been presented with its correct definition in this second session it was presented with the definition appropriate to its homophone and vice versa. For example, the patient is presented with the word "horca" (gallows) and its correspondent definition "where people were hung", and in another presentation, the same word "horca" but with a definition that corresponds to its homophone, "orca" (killer whale). Each item is considered correct when the subject accepts the correct definition and rejects the incorrect one.

M.B. correctly accepted and rejected 15/28 (54%) items for this homophone comprehension task. His low performance is not significantly different from chance level ($\chi^2$ (1) = 0.02, $p = 0.9$). Again, his performance in this task seems to confirm a central semantic impairment explanation for M.B.'s deficits.

*3.3.6. Irregular word-picture matching task*

Even though there is strong evidence in support of a central semantic deficit to account for M.B.'s performance in the semantic tasks, it could be argued that semantic information could have been preserved, exceptionally, for those IWs M.B. read correctly, and that that semantic knowledge might have helped M.B. to read the IWs correctly [40,41,53]. In order to test whether M.B.'s access to the semantic knowledge for the irregular words he read correctly was preserved or impaired, we designed a word-picture matching task with the same 40 irregular words from the reading list.

The target word was printed in the centre of the page and the patient had to choose between four alternatives (the target picture and three semantically related pictures, all of them from the same semantic category; for example, all hot beverages for the item "cappuccino").

M.B. correctly matched 8/40 words (20%) with their corresponding pictures, placing his score significantly below that of normal controls (mean score = 38; $\chi^2$ (1) = 46.04, $p < 0.001$). He correctly matched 6/20 (30%) words from the high familiarity group and 2/20 (10%) from the low familiarity group, though this difference did not reach statistical significance ($\chi^2$ (1) = 2.5, $p = 0.12$). These scores are very low, thus, M.B.'s performance in this matching task seems to reflect his difficulty in understanding the irregular words he can read correctly.

Table 3 shows M.B.'s and controls' performance in the semantic tasks. In summary, the results showed impaired access to semantic knowledge from different inputs: pictorial (from the picture naming task), written (from the rest of the tasks), pictorial and written (written word-picture matching task), and pictorial and spoken (spoken word-picture matching task). Thus M.B. showed semantic impairment regardless of the access modality, suggesting a semantic impairment [30,20].

## 4. Discussion

We have presented the case of a semantic dementia patient speaking a language, Rio de la Plata Spanish, with a predominantly transparent orthography who could read non-words, regular and irregular words despite his impaired semantic knowledge. The results from the visual word recognition tasks suggest that M.B.'s orthographic lexicon was unimpaired. Evidence for lexicality and frequency effects was found in the lexical decision with pseudohomophones task, further implicating the preserved functional importance of lexical knowledge in orthographic processing. On the other hand, the results from the semantic tasks that explored semantic knowledge and its different modalities of access showed impaired performance compared to control subjects. That pattern strongly suggests a semantic impairment as an explanation for M.B.'s deficit in semantic tasks. Additionally, and most striking, the patient showed semantic impairment of the regular and irregular words he could correctly name. These results have direct implications for the role of semantics in reading aloud irregular words. It seems to be the case that patient M.B. can read irregular words despite impaired semantic knowledge of the very same words.

In other words, the route for reading irregular exception words would be the lexical route [8]. Normally, it would imply semantic activation (namely, the lexical semantic route). But M.B. suffered from semantic dementia, a disease that directly affects semantic knowledge with relative preservation of both input and output lexicons. As can be seen from the experimental assessment, all tasks tapping semantics were severely affected, suggesting that M.B.'s lexical semantic route is impaired.

The previous results showed that M.B. was able to read IWs without the help of semantic information. Since these words cannot be read through GPC rules without leading to regularization errors these results seem to indicate that MB could directly map printed words to their corresponding phonological forms. Therefore, to correctly read irregular words without understanding them, M.B. should have made use of a direct connection between the orthographic input lexicon and the phonological output lexicon.

This pattern of performance could easily be explained by the existence of a direct whole-word connection between the orthographic input and the phonological output lexicons (see Fig. 1, route 3) in Spanish, as claimed by Ferreres et al. [18]. Nevertheless, at least two alternative accounts should be ruled out before considering the whole-word lexical connection explanation for M.B.'s unimpaired IW reading. On the one hand, the two-route summation model [26,27, 29], and on the other hand, the lexical response bias explanation [20].

The summation model proposes two routes for reading: a sub-lexical route based on grapheme-phoneme correspondences and a lexical-semantic routes (Fig. 1, routes 1 and 2, respectively). According to this view, an association between comprehension and reading of irregular words should be found. The summation model predicts that irregular words for which meaning is par-

Table 3
M.B. and controls' performance on semantic tasks

| Tasks | MB Score | MB % | Controls Score | Controls % | Sign. |
|---|---|---|---|---|---|
| Picture naming 64SB | 18/64 | 28 | 61/64 | 95 | ** |
| Written word-picture matching (BEA) | 11/20 | 55 | 20/20 | 100 | * |
| Spoken word-picture matching (BEA) | 12/20 | 60 | 20/20 | 100 | * |
| Pyramids and Palm Trees – pictorial | 22/52 | 42 | – | – | – |
| Pyramids and Palm Trees – verbal | 20/52 | 38 | – | – | – |
| Picture semantic categorisation (64SB) | 50/64 | 78 | 64/64 | 100 | ** |
| Homophones comprehension | 15/28 | 54 | – | – | – |
| Irregular Word-Picture Matching | 8/40 | 20 | 38/40 | 94 | ** |

$^{*}p < 0.01$; $^{**}p < 0.001$.

tially preserved – as seems to be the case for M.B. if his performance on the Irregular Word-picture matching task is considered- may still be read correctly if information specifying the regularized pronunciation output from the sublexical route would summate with information output by the partially functional lexical-semantic route. If that were the correct description for M.B.'s reading, then the summation theory would predict that the IWs for which M.B. still had some semantic knowledge – that is to say, those words correctly matched with the target picture in the IW-picture matching task- would be the same IWs he could read correctly [20]. Conversely, those IWs for which he showed impaired semantic knowledge – that is, the words he failed to match with the target picture- should also be those words he failed to read correctly because no lexical-semantic activation should arrive at the phonological level to be summed to the sub-lexical regularized form of the IW. Contrary to this prediction, M.B. only failed to read one IW but failed to match the IWs to the target pictures in 32 trials (80% error). In other words, he could read almost all the words without understanding their meaning [28]. This evidence seems to rule out the summation hypothesis as a possible explanation of M.B.'s ability to read IWs almost perfectly.

The lexical response bias explanation [20] was developed to account for the co-occurrence of comprehension deficits and surface dyslexia in a patient, E.P., with irregular word reading impairment. When reading IWs, patient E.P. produced both an irregular and a regularized response to the target word, suggesting that both his lexical and sublexical routes were competing for the selection of the pronunciation. E.P. seemed to prefer the irregular response when she had some residual knowledge of the word. This "vague" semantic knowledge was assessed by means of a word-picture matching task in which two kinds of distractors were presented together with the target picture. The distractors were either related (i.e., from the same semantic category, for example, if the target word was an animal, the distractor picture was another animal) or unrelated (i.e., from a different category: an animal and a means of transport, for example). Each target word was presented twice: once in the related condition, and once in the unrelated one and the patient had to match the word with the target picture. Funnell noted that the E.P. could match above chance level the IWs she read correctly only when the distractor picture was not semantically related to the target. In other words, she performed above chance level only when at least some semantic knowledge was still available to activate the right semantic category. Funnell [20] proposed that comprehension could have a less direct influence on reading than that proposed by Hillis and Caramazza [26,27] in the summation hypothesis. Funnell claimed that even some vague knowledge of the meaning of a word could be enough to bias the selection in favor of the lexical (irregular) response. Otherwise, the sublexical bias would prevail and regularization errors should be found when reading IWs.

It is noteworthy that M.B. differed from patient E.P. at least in three aspects. Firstly, unlike E.P., M.B. never produced a regularized response for the IWs. This shows a clear distinction between both patients' strategies when reading IWs. Secondly, M.B.'s reading of IWs was not impaired relative to his reading of regular words ($\chi^2$ (1) $= 0.03$, $p > 0.1$), since for both word types his performance was at ceiling. In other words, E.P. showed a surface dyslexic pattern, whereas M.B. did not. Thirdly, M.B.'s reading of IWs did not deteriorate over time: A similar IW reading task with 30 items was administered to M.B. two years before the current experimental assessment and he achieved 28/30 (93%) correct responses, showing no significant differences with his reading performance on the new IW reading task ($\chi^2$ (1) $= 0.73$, $p > 0.1$). Contrarily, patient E.P. achieved 34/39 (87%) correct responses on the Coltheart's set and 23/39 (59%) correct responses two years later on the same set of words [20, p. 423], showing a significant deterioration in her performance over time ($\chi^2$ (1) = 7.88, $p < 0.01$). If in spite of the above mentioned differences between the two patients, the lexical bias hypothesis is an adequate account for M.B.'s reading, then he should be able to show some comprehension (i.e., in the word-picture matching task) of the words he read correctly but would fail to comprehend the only word he mispronounced in the reading task [20]. However, as we have already pointed out, he only misread one word but failed to comprehend 80% of the IWs.

Furthermore, Funnell claims that the evidence from patient E.P.'s IW reading suggests that the direct lexical non-semantic connections were not completely functional, otherwise the patient would have always selected the irregular correct pronunciation for IWs. Interestingly, that seems to be the case for M.B.'s performance: Except for one word, he always preferred the irregular pronunciation when reading irregular words.

In the light of this evidence, the lexical bias hypothesis could be ruled out as an explanation of patient M.B.'s pattern of impaired semantics and preserved IW reading. Thus, the notion of "wordness" – that is to say, the notion of a direct whole-word lexical connection- seems to be applicable for patient M.B., unlike patient E.P. Additionally, this concept of "wordness" seems to be further confirmed by the results of the IW phonology-orthography matching tasks. In this task, M.B. seems to directly map the irregular phonology onto the irregular orthography of these words, even though his semantic knowledge is impaired.

In conclusion, the case study presented here shows quite convincingly that reading irregular words can be done even with impaired semantics and in a language with a transparent orthography like Spanish. Our results provide evidence against the idea that the degree to which semantic knowledge is preserved impacts on reading performance, that is, against the idea that relative lexical semantic activation input to the phonological output lexicon would predict whether a competing sublexical alternative would prevail over the correct irregular pronunciation. Our evidence is consistent with an account in which performance on irregular word reading indicates the use of a direct whole-word lexical route in reading aloud even in a transparent orthography.

## Acknowledgments

We are grateful to Cristina Burani and Fernando Cuetos for their helpful comments on a previous version of the manuscript. Also, we thank Claudio Luzzatti for his kind help and an anonymous reviewer for his valuable suggestions and comments. We are also very grateful to M.B. and his family for their cooperation and patience.

## Appendix A

Irregular low (LF) and high (HF) familiarity words (and their familiarity means and standard deviations, SD) used in IW reading, word-picture matching, and phonology-to-orthography matching tasks.

| Familiarity group | | | | | |
|---|---|---|---|---|---|
| Low | | | High | | |
| | Familiarity | | | Familiarity | |
| Item | Mean | SD | Item | Mean | SD |
| Beeper | 2,46 | 1,72 | Baguette | 4,96 | 1,85 |
| Boite | 1,70 | 1,29 | Bijouterie | 4,92 | 1,91 |
| Bouquet | 1,83 | 1,27 | Bowling | 4,50 | 2,00 |
| Boutique | 3,50 | 2,34 | Brownie | 4,83 | 1,69 |
| Bridge | 2,50 | 1,67 | Cappuccino | 5,96 | 1,08 |
| Cognac | 3,25 | 1,75 | Champignon | 4,54 | 2,38 |
| Crepe | 2,88 | 1,92 | Crochet | 4,13 | 2,07 |
| Croissant | 3,38 | 1,95 | Freezer | 6,25 | 1,36 |
| Croupier | 2,54 | 1,91 | Garage | 5,83 | 1,55 |
| Croûton | 2,04 | 1,40 | Jean | 6,21 | 1,32 |
| Iceberg | 3,21 | 1,77 | Lasagna | 4,42 | 1,93 |
| Mignon | 3,08 | 2,00 | Omelette | 5,17 | 2,08 |
| Panaché | 2,38 | 2,00 | Pool | 5,38 | 1,53 |
| Parchet | 2,71 | 1,78 | Porche | 4,79 | 1,96 |
| Referee | 2,88 | 2,07 | Shampoo | 6,54 | 0,93 |
| Roller | 3,38 | 1,95 | Sommier | 5,21 | 1,35 |
| Soutien | 2,71 | 2,10 | Souvenir | 4,71 | 2,12 |
| Vermouth | 3,17 | 1,83 | Sweater | 5,29 | 1,76 |
| Vitreaux | 3,50 | 2,38 | Toilette | 4,42 | 2,12 |
| Voucher | 3,50 | 2,21 | Vedette | 4,42 | 1,64 |
| *LF Group* | *2,83* | *0,56* | *HF Group* | *5,12* | *0,70* |
